# Complete Genome Sequence of *Riemerella anatipestifer* Serotype 1 Strain CH3

**DOI:** 10.1128/genomeA.01594-14

**Published:** 2015-02-19

**Authors:** Xiaolan Wang, Chan Ding, Xiangan Han, Shaohui Wang, Jiaping Yue, Wanwan Hou, Shoulin Cao, Jiechi Zou, Shengqing Yu

**Affiliations:** Shanghai Veterinary Research Institute, Chinese Academy of Agricultural Sciences, Shanghai, China

## Abstract

*Riemerella anatipestifer* is a well-described pathogenic bacterium, which is reported worldwide as the cause of epizootic infectious polyserositis of waterfowl and other avian species. Here, we present the complete genome sequence of *R. anatipestifer* strain CH3, the serotype 1 prevalent in China.

## GENOME ANNOUNCEMENT

*Riemerella anatipestifer* is a Gram-negative rod-shaped bacterium which was first reported by Riemer as a pathogen of geese (1). It has been isolated from all kinds of avian hosts, including ducks, turkeys, chickens, and other birds (2, 3). In domestic ducks, *R. anatipestifer* causes epizootic infectious polyserositis, characterized by lethargy, diarrhea, and respiratory and nervous symptoms, which lead to high mortality and consequently to great economic losses (4). Currently, 21 serotypes of *R. anatipestifer* have been identified, and no significant cross-protection has been reported (5). Infections by *R. anatipestifer* serotype 1, 2, 3, 5, 6, 7, 8, 10, 11, 13, 14, and 15 strains have been reported in China, with serotypes 1, 2, and 10 responsible for most of the major outbreaks (6). There have been several genomic resources available for *R. anatipestifer* (7, 8). Here, we report another complete genome sequence of *R. anatipestifer* serotype 1 strain CH3.

The complete genomic sequencing was conducted using a Roche GS FLX system (9). A total of 186,353 reads totaling 70,430,692 bases (average read length, 377 bp) was obtained, resulting in 11-fold genome coverage. Newbler software (version 2.3) (Roche) was used for sequence assembly. Sequence gaps were filled in by first determining the order of contigs using multiplex PCR (10) and then sequencing the PCR products using ABI 3730xl capillary sequencers. Low-quality regions of the genome were resequenced to obtain the complete linear genome sequence. The final sequencing accuracy was 99.99%. Putative coding sequences (CDS) were identified by Glimmer 3.02 (11) and the Z-Curve program (12). Transfer RNA genes were predicted by tRNAscan-SE (13), while rRNAs were predicted by RNAmmer (14). Functional annotation of CDSs was performed through searching against the nr protein database using BLASTP (15). Clusters of orthologous group (COG) function classifications were performed using the CDD database (http://www.ncbi.nlm.nih.gov/cdd) (16). Orthologs and paralogs were defined as proteins with greater than 30% similarity. The metabolic pathways were constructed using the KEGG database (http://www.genome.jp/kegg/).

*R. anatipestifer* strain CH3 possesses a single circular chromosome of 2,234,477 bp with a coding percentage of 90.0%. The genome contains 2,186 putative open reading frames (ORFs) with an average length of 920 bp, 9 rRNA operons, and 41 tRNA genes. Of the ORFs, 2,041 were protein-coding genes. The majority of the protein-coding genes (1,359 [66.59%]) were classified into the COG families comprising 20 functional categories. Using KEGG, 908 ORFs (44.49%) were assigned to 30 pathways.

Among all proteins, 16 proteins involved in peptidoglycan biosynthesis and 12 proteins involved in lipopolysaccharide (LPS) biosynthesis were identified. LPS, which functions as an endotoxin, an adhesin, and a modulator of the immune response, is an important virulence factor for numerous bacteria (17–19). The genome sequence provides essential information on understanding the molecular pathogenesis and protective mechanism of *R. anatipestifer*.

### Nucleotide sequence accession number.

This whole-genome shotgun project has been deposited in DDBJ/ENA/GenBank under the accession no. CP006649. The version described in this paper is the first version.
